# 

**Published:** 1907-04

**Authors:** 


### The American Journal of Dental Science

The Official Organ of the Florida, Georgia and South Carolina State Dental Societies

Third Series

VOL. XXXVIII APRIL, 1907 NO. 4

EDITORS

WM. GIRD BEECROFT, D. D. S., Madison. Wis. B. H. TEAGUE, D. D. S., Aiken, S. C.

Published on the 10th day of each month.
Subscription price $1.00 per year.
ENTERED AS SECOND-CLASS MATTER, MADISON, WIS.

Address all communications, both business and editorial, to
The Dental Publishing Company, Madison, Wisconsin

